# Linking Pesticide Exposure with Pediatric Leukemia: Potential Underlying Mechanisms

**DOI:** 10.3390/ijms17040461

**Published:** 2016-03-29

**Authors:** Antonio F. Hernández, Pablo Menéndez

**Affiliations:** 1 Department of Legal Medicine and Toxicology, University of Granada School of Medicine, Granada 18016, Spain; 2 Department of Biomedicine, Josep Carreras Leukemia Research Institute, School of Medicine, University of Barcelona, Barcelona 08036, Spain; 3 Instituciò Catalana de Recerca i Estudis Avançats (ICREA), Barcelona 08010, Spain

**Keywords:** infant and childhood leukemia, hematopoietic stem/progenitor cells, chromosomal rearrangements, topoisomerase II, pesticides, DNA double-strand break, oxidative stress

## Abstract

Leukemia is the most common cancer in children, representing 30% of all childhood cancers. The disease arises from recurrent genetic insults that block differentiation of hematopoietic stem and/or progenitor cells (HSPCs) and drives uncontrolled proliferation and survival of the differentiation-blocked clone. Pediatric leukemia is phenotypically and genetically heterogeneous with an obscure etiology. The interaction between genetic factors and environmental agents represents a potential etiological driver. Although information is limited, the principal toxic mechanisms of potential leukemogenic agents (e.g., etoposide, benzene metabolites, bioflavonoids and some pesticides) include topoisomerase II inhibition and/or excessive generation of free radicals, which may induce DNA single- and double-strand breaks (DNA-DSBs) in early HSPCs. Chromosomal rearrangements (duplications, deletions and translocations) may occur if these lesions are not properly repaired. The initiating hit usually occurs *in utero* and commonly leads to the expression of oncogenic fusion proteins. Subsequent cooperating hits define the disease latency and occur after birth and may be of a genetic, epigenetic or immune nature (*i.e.*, delayed infection-mediated immune deregulation). Here, we review the available experimental and epidemiological evidence linking pesticide exposure to infant and childhood leukemia and provide a mechanistic basis to support the association, focusing on early initiating molecular events.

## 1. Introduction

Leukemia is the most common childhood cancer, accounting for 30% of all cancers diagnosed in children under 15 years of age, with an annual incidence of up to 40 cases per million children in developed countries and an incidence peak between three and five years of age [1,2]. Pediatric acute leukemia is a phenotypically- and genetically-heterogeneous disease of immature hematopoietic stem and progenitor cells (HSPCs). Phenotypically, it can target B-cell progenitors (B-cell acute lymphoblastic leukemia (B-ALL)), T-cell progenitors (T-ALL) or myeloid progenitors (acute myeloid leukemia (AML)). Acute leukemia can be further stratified according to the differentiation stage at which HSPCs are blocked; for example, B-ALL can have a pro-B (proB-ALL) or pre-B phenotype (preB-ALL) [3]. Similarly, AML can affect both immature (subtype M0 of the French-American-British classification of AML) and mature lineage-committed types, such as erythroblastic or megakaryoblastic leukemia (subtypes M6 and M7, respectively). Seventy percent of pediatric acute leukemias are ALL and 30% are AML. Genetically, ALL and AML can be further stratified according to molecular cytogenetics [4,5], which represents a prognostic factor.

Fetal hematopoiesis begins in the aorta gonad-mesonephros region to subsequently colonize the fetal liver (FL) and ultimately, just before birth, the bone marrow [6]. FL hematopoiesis entails an active proliferation of progenitors, rendering fetal HSPCs susceptible to oncogenic transformation through DNA damage mediated by chemical exposure during pregnancy [7]. Although the etiology of ALL remains elusive, ionizing radiation, congenital genetic syndromes and *in utero* exposure to specific genotoxic chemicals, including household pesticides, represent prime etiological suspects [8]. Importantly, altered patterns of infection during early childhood might also contribute to acute leukemia in children [9,10,11].

We here review the available experimental and epidemiological evidence linking pesticide exposure with infant and childhood leukemia and provide a mechanistic basis to support the association, focusing on early molecular events. However, the paucity of mechanistic data is a major obstacle to fully understanding the toxicological pathways involved. Causation pathways are likely to be multifactorial, and it is possible that the risk of pediatric leukemia from environmental exposure is influenced by genetic susceptibility.

## 2. Evidence Linking Pesticide Exposure with Pediatric Leukemia

### 2.1. Epidemiological Studies Supporting the Association

There is a growing concern about whether chronic low-level pesticide exposure during pregnancy or childhood increases the risk of childhood leukemia. Epidemiological studies suggest that pesticide exposure may have a greater impact on children than adults [12,13]. Almost all of the available evidence has focused on pediatric leukemia without making a distinction between infant and childhood leukemia, which are etiologically and pathologically different entities. However, most epidemiological studies are limited because no specific pesticides have been directly associated with the risk of leukemia, but rather the broad term “pesticide exposure” [13,14]. Such associations are mainly based on subjects’ recall of the pesticide exposure, which hampers the drawing of conclusions because of recall/information bias.

In contrast to childhood leukemia, very few studies have examined the risk of infant leukemia and pesticide exposure. An international collaborative study on transplacental chemical exposure and risk of infant leukemia found an increased risk after *in utero* exposure to household pesticides (propoxur and other methylcarbamate insecticides), the therapeutic analgesic dipyrone and hormonal intake (estrogens). In these cases, infant leukemia was associated with the mixed lineage leukemia (*MLL*) gene fusion, likely as a result of topoisomerase II inhibition [15,16]. Although the aforementioned study was based on a rather small sample size, an increased risk (Odds Ratio—OR: 2.18) of infant leukemia was shown in mothers exposed to domestic insecticides during pregnancy. Since estrogens can be metabolized to catechol estrogen-3,4-quinones [17], the association found for infant leukemia might be due to topoisomerase II inhibition caused by quinone metabolites generated during estrogen metabolism [7]. A further Brazilian study found that over use of pesticides during pregnancy was associated with ALL and AML (OR: 2.10 and 5.01, respectively) in children <1 year of age [18]. Moreover, maternal exposure to the insecticide permethrin (assessed by self-reporting) was associated with a higher risk of leukemia in children <1 year of age, with an OR of 2.47 for ALL and 7.28 for AML. This finding was also supported by a case-control study in China where the use of pyrethroids (assessed by urine levels of major metabolites) was associated with a greater risk of ALL [19].

The presence of the herbicide chlorthal in household dust samples was also associated with an increased risk of ALL in children <8 years, with a significant dose-response trend [20]. The association was greater with the herbicide mixture chlorthal plus alachlor. Other studies, however, report no significant associations. For example, no significant risk of childhood leukemia was found with exposure to some agricultural and residential herbicides, such as metolachlor, bromoxynil, cyanazine and 2,4-dichlorophenoxyacetic acid [20,21]. Furthermore, a case-control study on leukemia in children <1 year old from the American Children’s Oncology Group failed to find a significant association between household exposure to insecticides or rodenticides and ALL or AML [22].

Different meta-analyses have consistently shown an increased risk of childhood leukemia associated with pesticide exposure [13,23]. However, this review will focus on the latest quantitative synthesis of evidence from studies. A recent meta-analysis has shown that maternal occupational pesticide exposure during pregnancy and/or paternal occupational pesticide exposure near-to-conception increases the risk of leukemia in offspring [24]. The authors pooled data from 13 case-control studies participating in the Childhood Leukemia International Consortium (CLIC) and found an almost two-fold increased risk of AML in mothers exposed to pesticides during pregnancy, whereas no significant risk was found for paternal exposure around conception. In relation to ALL, the same study observed a 20% increased risk with paternal exposure around conception, which appeared to be more evident for pediatric T-cell ALL. By contrast, no significant association was found between maternal exposure during pregnancy and risk of B or T-cell ALL. In a separate study investigating residential pesticide exposure, Bailey *et al.* [25] pooled data from 12 case-control studies in the CLIC and found a significant increased risk of ALL associated with exposure to any pesticide shortly before conception, during pregnancy and after birth (OR: 1.39, 1.43 and 1.36, respectively). Little variation was observed with the type of pesticide. Regarding AML, an increased risk was found for exposure to any pesticide in the few months prior to conception and during pregnancy (OR: 1.49 and 1.55, respectively); however, exposure after birth failed to demonstrate an increased leukemogenic risk. A recent meta-analysis conducted by Chen *et al.* [12] pooled 16 case-control studies and found that childhood exposure to indoor, but not outdoor, residential insecticides was associated with an increased risk of pediatric leukemia (OR: 1.47). A slightly weaker association was found for herbicide exposure (OR: 1.26). Notwithstanding these positive associations, observational studies on pesticide exposure and pediatric leukemia have a number of weaknesses to claim causal relationships. The consistency of findings across meta-analyses may be due to the considerable overlap in the studies included in the different meta-analyses undertaken. Many epidemiological analyses have not been performed using methodologically-rigorous association studies. Limitations include the lack of an accurate exposure estimate (from both a qualitative and quantitative standpoint), lack of temporal concordance (most studies were case-control in design) and little information on the dose-response relationship. In addition, the available epidemiological evidence may be challenged by endogenous or exogenous factors, such as genetic susceptibility, lifestyle and co-exposure to other environmental agents.

### 2.2. In Vitro Studies

The few *in vitro* studies available so far have shown that captan and captafol (two related chloroalkylthiocarboximide fungicides) decrease the activity of topoisomerase II by 50% and 20%, respectively, at a concentration of 1 µM [26]. Similarly, thiram (a dithiocarbamate fungicide) inhibits topoisomerase II at 10 µM [27]. However, genotoxic potential (*i.e.*, genetic abnormalities, mutations) of these fungicides occurred only at very high doses (10–100 mM) *in vivo* using common fruit flies [26]. More recently, the organophosphate (OP) insecticide chlorpyrifos has been reported to induce DNA double-strand breaks (DSBs) and *MLL* gene rearrangements in human fetal liver CD34^+^ HSPCs as a consequence of topoisomerase II inhibition [14].

Other OP pesticides have been implicated in leukemogenesis, particularly isofenphos, diazinon and fenitrothion. An *in vitro* study using the human leukemic cell line K562 demonstrated metabolic changes consistent with a leukemogenic potential of isofenphos [28]. In addition, human peripheral blood lymphocytes exposed to isofenphos exhibited dose-dependent damage to chromosomal DNA, as well as disruption of the cholinergic nuclear signaling pathway, which collectively could lead to genomic instability and leukemogenesis [29]. In an *in vitro* study using diazinon, a concentration of 0.1 µM induced hypermethylation of several genes involved in cell cycle arrest, such as cyclin-dependent kinase inhibitor 1A (*CDKN1A*) and 1C (*CDKN1C*), and tumor suppressor genes, such as *p53* and *PTEN* [30]. Fenitrothion at low concentrations (1 µM) also induced chromosomal damage in the B-cell leukemia/lymphoma-2 cell line BCL-2 [31].

## 3. Gene-Environment Interactions

For most pediatric leukemias, multiple genetic polymorphisms of xenobiotic metabolizing enzymes may interact with environmental, dietary and maternal factors to modulate the development of the disease. For example, quinones, which are capable of inhibiting topoisomerase II and can cleave the *MLL* gene at topoisomerase II cleavage sites, may be poorly detoxified depending on the activity of NAD(P)H:quinone oxidoreductase 1 (NQO1), an enzyme that detoxifies chemicals with quinone rings, such as bioflavonoids and benzene metabolites. Thus, genetic polymorphisms of *NQO1* resulting in low-activity variants might be associated with an increased risk of infant leukemia. By contrast, in childhood ALL without *MLL* rearrangements, deficiency of the *NQO1* gene is not associated with the etiology of the disease [32].

Global DNA hypomethylation is associated with activation of oncogenes and neoplastic processes [33], whereas the hypermethylation of 5′ cytosine-phospho-guanine (CpG) islands in promoter regions of some tumor suppressor genes prevents their transcription and promotes the development of tumors [34]. The genetic regulation of folate metabolism may have an influence on the preleukemic clone origin via DNA hypomethylation of key regulatory genes, rendering the genome vulnerable to genomic instability [35]. The presence of some polymorphisms in genes involved in folate metabolism reduces enzyme activity, leading to inadequate folate levels and DNA hypomethylation, ultimately contributing to the neoplastic process [35,36]. The insufficient input of folate increases the plasma concentration of homocysteine and *S*-adenosylhomocysteine, with the latter being a general inhibitor of adenosylmethionine-dependent methyltransferases [37]. Inhibition of these enzymes may alter both DNA methylation and transcriptional regulation [36,38]. The 677C>T gene polymorphism in methylenetetrahydrofolate reductase (*MTHFR*) has been linked to a decreased risk of childhood ALL, likely as a result of higher production of 5,10-MTHF and thymidine, which improve the fidelity of DNA synthesis and repair [39]. On the other hand, inactivating polymorphisms of detoxifying enzymes involved in carcinogen metabolism, such as glutathione *S*-transferases (GST), in parents have been associated with the development of ALL in their children <1 year old. The deletion of both the *GSTT1* and *GSTM1* genes in either parent might affect the risk of infant leukemia [40]. Furthermore, genetic polymorphisms of xenobiotic transport and metabolism pathways are associated with the risk of childhood ALL. In particular, polymorphisms of the *ABCB1* gene, which encodes a membrane transporter of lipophilic compounds, may interact with household insecticide exposures to increase the risk of disease [41]. Genetic variability in DNA repair pathways and cell cycle checkpoints might also interact with environmental, dietary, maternal and other external factors affecting the development of ALL. In summary, the limited data available suggest that dietary and environmental exposure to substances targeting topoisomerases together with the reduced ability of fetuses or their mothers to detoxify such compounds because of polymorphic variants of given genes could contribute to the development of pediatric leukemia [8,42].

The International Childhood Acute Lymphoblastic Leukemia Genetics Consortium revealed limitations in current studies on genetic susceptibility and the risk of ALL because of difficulties in conducting statistically- and methodologically-rigorous investigations [43]. Genome-wide association studies of childhood ALL have provided robust evidence for four low-penetrance susceptibility variants, which confer only a modest increase in risk. Moreover, the well-recognized ethnic differences in the risk of ALL represent a weakness in assessing the interplay between inherited and non-genetic risk factors. Given the small frequency of many ALL subgroups, the identification of differential effects will realistically be possible only through multi-center pooled analyses [43].

## 4. Early Molecular Events Involved in Pesticide-Associated Pediatric Leukemogenesis

Despite the rather comprehensive epidemiologic evidence linking pesticide exposure during different reproductive stages (pre-conception, pregnancy and early postnatal life) and pediatric leukemia, robust underlying pathological mechanisms remain unknown. The initiating event at the molecular level might be the induction of chromosomal rearrangements as a result of pesticide exposure and subsequent topoisomerase II inhibition or generation of oxidative stress, leading directly or indirectly to DNA damage. A mechanistic explanation follows.

### 4.1. DNA Double-Strand Breaks (DSBs)

Under some circumstances, oxidative lesions can lead to DNA DSBs formation in HSPCs. Environmental exposures to numerous chemicals, including many pesticides, have been shown *in vivo* and *in vitro* to generate oxidative species that can ultimately induce DNA base or sugar oxidative damage, leading to single-strand breaks (SSBs) and DSBs formation in the DNA [44]. For example, OP insecticides (chlorpyrifos, methyl-parathion, malathion), methyl-carbamates (methomyl) and the herbicide paraquat all cause oxidative DNA damage followed by DNA SSBs and DSBs [45,46,47,48]. There is also evidence of pesticide-induced oxidative stress and DNA damage in agricultural workers [47]. Additionally, oxidative species may interact with biological molecules to disrupt normal DNA synthesis and repair, and so, inhibition/inactivation of antioxidant proteins or DNA repair enzymes may also be an underlying molecular mechanism [49]. Along this line, pesticides can disrupt a number of antioxidant enzymes, including superoxide dismutase and catalase [50], rendering oxidative stress [51].

DSBs can arise under different circumstances: (i) when two SSBs form close to each other on opposite strands; (ii) upon enzymatic DNA cleavage next to an SSB on the opposite strand; or (iii) when either DNA replication or transcription takes place at sites of misrepaired DNA. DSBs constitute the first molecular event in the generation of chromosomal aberrations [52]. For instance, chlorpyrifos is reported to cause DNA DSBs and further chromosomal rearrangements (*i.e.*, *MLL*) through oxidative stress in human FL HSPCs [53]. However, chlorpyrifos can also induce DNA DSBs as a result of topoisomerase II inhibition in FL HSPCs in a manner similar to that produced by etoposide [14]. Analogously, blood lymphocytes from pesticide sprayers have greater fragile site breakage than normal individuals following treatment with aphidicolin, an inhibitor of DNA polymerases [54]. Chromosomal fragile sites are regions of the genome prone to breakage following exposure to many chemicals, including environmental and chemotherapeutic agents. During DNA replication, fragile site-inducing conditions can uncouple the helicase complex from the DNA polymerase, resulting in long stretches of single-stranded DNA and further DNA breakage [55]. Aphidicolin can also induce fragile site breakage through a topoisomerase II-mediated mechanism [56].

Topoisomerase II has critical functions in both DNA replication and transcription processes, and the so-called “topoisomerase II poisons” disrupt the DNA-induced topoisomerase II cleavage-religation equilibrium through the stabilization of ternary (drug-DNA-enzyme) complexes, termed cleavage complexes [57]. Chemical-induced breakpoints are strongly associated with predicted topoisomerase II cleavage sites (*i.e.*, *MLL*), thus supporting a role for topoisomerase II-mediated breakage upon exposure to environmental agents. The high frequency of topoisomerase II recognition sites in specific DNA regions and the high expression of this enzyme in human CD34^+^ HSPCs represent favorable conditions for breakage following exposure to agents targeting topoisomerase II activity (*i.e.*, bioflavonoids and quinones). Because CD34^+^ HSPCs appear to be more sensitive to DNA damage than committed progenitor cells, exposure to low levels of different chemicals may induce DNA breakage at certain sites in HSPCs, increasing the risk of chromosomal rearrangements. If affected cells survive, they continue growing and dividing, thus perpetuating DNA lesions and starting the chain of events that will eventually lead to leukemogenesis [55].

### 4.2. Chromosomal Translocations

Key molecular events leading to pediatric leukemia pathogenesis are chromosomal translocations. These generally result from the exchange of chromosomal arms between heterologous chromosomes, and DNA DSBs are prerequisites for their occurrence. Chromosomal translocations ultimately result in the deregulation of key cellular proteins, especially those encoded by proto-oncogenes and tumor suppressor genes, which are critical functional regulators of the cell [58]. Two functional classes of translocations are known. The first one relocates a proto-oncogene (or genes encoding for non-antigen receptors or transcription factors) into regulatory regions of actively-transcribed genes (such as those encoding for immunoglobulin chains or T-cell receptors), causing dysregulated expression of an intact protein. The second class of translocations juxtaposes two genes to encode a chimeric protein, which is functionally distinct from the wild-type proteins [1].

Although the mechanistic generation of chromosomal translocations is not well understood, they may arise from improper DNA repair or erroneous recombination of variable (V), diversity (D) and joining (J) gene segments (a process known as V(D)J recombination). As for improper DNA repair, reactive oxygen species (ROS)-induced DSBs in human FL CD34^+^ HSPCs following maternal exposure to chemicals triggers recombination/repair pathways by non-homologous end-joining (NHEJ) [14]. The majority of damaged HSPCs may either successfully repair the DNA DSBs or fail to do so and undergo apoptotic cell death. In a fraction of cells, the repair of the DNA DSBs within particular breakpoint cluster regions (bcr) is not completed correctly, giving rise to chromosomal translocations or deletions [59]. For fusion genes to be leukemogenic, DSBs must occur simultaneously in two chromosomes and must also involve the coding region of the genes to generate an exon-exon in-frame functional chimeric gene product. Importantly, this has to occur in an HSPC that has managed to bypass cell death and displays a sustainable lifespan and clonal potential to propagate the chimeric gene product [60].

Erroneous V(D)J recombination usually occurs in developing lymphocytes during cell maturation, where V(D)J gene segments of immunoglobulin chains or T-cell receptors are rearranged to yield a wide range of immunoglobulins and T-cell receptors. The process entails the cleavage of the V(D)J gene at the flanking recombination signal sequences (RSS) by lymphocyte-specific recombination-activating gene (RAG) endonucleases and subsequent ligation of the segments via the classical NHEJ pathway [61]. In pediatric leukemia, chromosomal translocations and deletions often arise as a result of mistakes in V(D)J rearrangements because RAG enzymes can erroneously recognize and target RSS-like sequences. V(D)J-recombinase-mediated rearrangements may occur at both immune RSS and non-immune cryptic RSS (cRSS), which are widely distributed throughout the genome [62]. There is growing evidence that *in vivo* exposure to DNA-damaging agents, including pesticides, can increase the frequency and alter the recombination site distribution of V(D)J rearrangements at cRSS [63,64]. An increase in V(D)J-recombinase-mediated events at either immune or non-immune RSS following exposure to DNA-damaging agents could play an important role in environmentally-induced genetic alterations associated with leukemia development. Nonetheless, the mechanism by which exposure to DNA-damaging agents could increase the frequency of V(D)J-recombinase-mediated genomic rearrangements remains unclear [64].

## 5. Pathobiology of Pediatric Leukemias

Given the distinct natural history and pathogenesis of infant and childhood leukemia, both entities will be addressed separately, although a chromosomal translocation is frequently the common initiating oncogenic event in both entities.

### 5.1. Infant Leukemia

**Figure 1:**
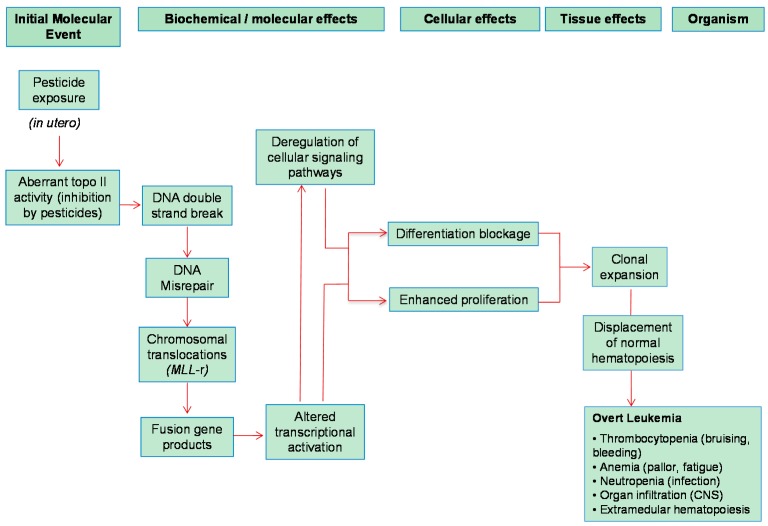
Chain of pathogenic events linking pesticide exposure to the development of infant *MLL*-rearranged acute leukemia.

Infant acute leukemia shows unique clinical and biological features and is commonly associated with rearrangements in the *MLL* gene (*MLL*-r), a master gene located on chromosome 11q23 that regulates normal human hematopoietic development and differentiation [65]. The *MLL* gene encodes a methyltransferase with activity for lysine 4 of histone H3 (H3K4), which mediates changes in chromatin associated with epigenetic transcriptional activation that plays an essential role in regulating gene expression during early development and hematopoiesis [66]. Rearrangements involving the *MLL* gene have been reported to occur only in mice with defects in DNA damage response and not in wild-type animals [67]. *MLL*-r functions as the initiating, and perhaps the sole driving, oncogenic event by dysregulating epigenetic and/or transcriptional programs [33] (Figure 1). Epidemiological and genetic studies have suggested that *MLL*-r may result from transplacental exposure to DNA topoisomerase-II inhibitors during gestation, such as chemotherapeutic agents, benzene metabolites (*i.e.*, benzoquinone), quinolone antibiotics, bioflavonoids present in some fruits and vegetables and some pesticides [7,33,68]. However, exposure to topoisomerase-II inhibitors is not sufficient *per se* for rearrangement of *MLL*, and the genetic background, such as mutations in the DNA damage response pathway, may influence the likelihood of *MLL*-r [67].

The existence of recombination-prone sequences in the *MLL* bcr region supports the contention that *MLL*-r results from DNA breakage and recombination events. The genomic instability within *MLL* bcr may be the consequence of increased ROS generation [69]. The *MLL* fusion gene renders HSPCs more vulnerable to DNA repair and cell-cycle deregulation, facilitating the rapid acquisition of additional, secondary genetic changes, particularly upon continued exposure to genotoxic chemicals *in utero* [7,70]. These chemicals target early mesodermal precursors or HSPCs residing mainly in the FL where they inhibit topoisomerase-II activity and produce DNA DSBs within the *MLL* bcr, which are not properly repaired by homologous recombination or NHEJ. Because those mesodermal precursors or HSPCs are rapidly dividing and have high topoisomerase II content, they may be particularly sensitive to damage by topoisomerase II-targeting chemicals during a critical developmental window of vulnerability [33,71,72,73,74,75]. However, because of the very short latency of infant leukemia, it remains obscure whether the fusion gene generated from chromosomal translocations requires additional cooperating oncogenic hits for leukemogenesis. Although recurrent activating mutations of genes associated with cellular proliferation, such as components of the RAS signaling pathway, have been reported [76,77,78,79], functional studies revealed that these mutations are important for tumor maintenance rather than initiation in human HSPCs [80]. *MLL* breakage itself is not sufficient for the development of full-blown infant leukemia, even if the DNA damage response is defective. Activation of cellular proliferation by mutation of other genes might be necessary for overt leukemia [67]. The transformation mediated by the aberrant proteins encoded by fusion genes might depend on alternative (epi)-genetic cooperating lesions at a critical developmentally-earlier window of stem cell vulnerability to develop overt leukemia [33].

Intriguingly, and in contrast to the global dogma of cancer biology, *MLL*-r infant leukemia has been shown to have abnormal hypermethylation in non-enhancer, non-promoter regions, perhaps contributing to genomic stability and a silenced mutational landscape [76,81,82]. Extensive hypermethylation of tumor suppressor genes resulting in gene silencing has been observed in some cases of *MLL*-r infant leukemia [83].

### 5.2. Childhood Leukemia

Childhood leukemia has a prevalence peak at ~3–5 years of age, suggesting that environmental exposures *in utero* or during early childhood might be risk factors [25]. Under the current paradigm, the first initiating oncogenic mutation usually involves structural or numerical chromosomal alterations, impairing normal cell differentiation, while secondary hits more commonly comprise mutations affecting developmentally-regulated master transcription factors or membrane-proximal signaling pathways conferring proliferation and survival advantages to the differentiation-blocked clone [1,7,8,84,85]. The development of leukemia requires the activation of cell proliferation in addition to differentiation blockage [67]. Numerical aberrations (*i.e.*, hyperdiploidy) are also common hallmarks in childhood B-cell ALL.

**Figure 2:**
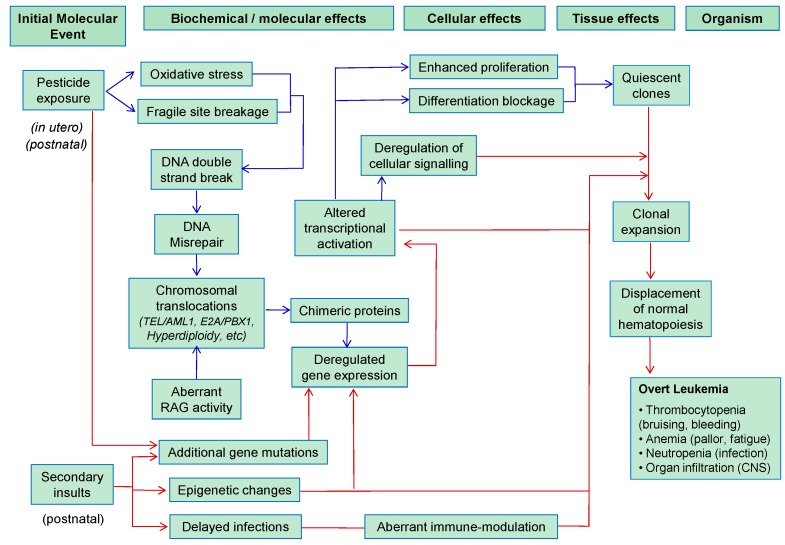
Chain of pathogenic events linking pesticide exposure to the development of childhood leukemia. Blue arrows indicate events related to the “first hit” and red arrows events related to the “second hit” (for more details, see Section 5.2).

The most common chromosomal aberrations are *E2A-PBX1*, *TEL-AML1* and *MLL*-r for B-ALL and *AML1-ETO* and *MLL*-r for AML. Similar to *MLL* rearrangements, the resulting aberrant chimeric proteins alter the normal transcriptional program and block normal B-cell and/or myeloid differentiation [8,86,87,88] (Figure 2). Although the *AML1* gene has been linked to anti-topoisomerase II agents, similar to the *MLL* gene, *TEL-AML1* is not sufficient to cause the disease by itself. As this fusion gene is observed in cord blood from about 1% of normal newborns, a significant proportion of the population carries self-limiting preleukemic clones, and the majority of them do not result in disease [3]. The longer latency observed in childhood leukemia unequivocally indicates that the initiating chromosomal translocation itself is unlikely to convert a preleukemic clone into an overt disease, thus suggesting the need for secondary cooperating (epi)-genetic events.

Dysfunction of the immune system and delayed infections have been linked to childhood leukemia [9,89]. Two distinct underlying mechanisms might explain this association: (i) a lower repertoire of infections during early immune development; and (ii) an altered congenital responder status to infection resulting in functionally-aberrant clinical presentation of occasional infections. Thus, an untimely and excessive inflammatory response abolishes normal hematopoiesis, promoting selective expansion of a preleukemic clone (Figure 2) because of proliferative advantage and increased likelihood for a second mutation required for the development of the disease to occur [33]. In turn, early childhood infections or vaccination may reduce the likelihood of leukemia [90]. Importantly, the major histocompatibility genes might play a role in the linkage between patterns of infection and leukemia risk, as several HLA haplotypes have been associated with childhood leukemia [3]. However, other studies have suggested that major histocompatibility complex-defined variation in immune-mediated response is unlikely to be a major risk factor [91].

Aberrant RAG activity resulting in genomic rearrangements may be a crucial secondary mechanism leading to B-cell ALL. Aberrant RAG activities can result in various oligoclonal V(D)J recombination events and the inactivation of genes required for B-lineage differentiation [87]. A clear link between RAG and childhood leukemia through inflammatory mechanisms has been recently reported [89], further connecting immune system-RAG-childhood leukemia.

## 6. Role of Acetylcholinesterase in Leukemogenesis

Moderate acetylcholine (ACh) levels are crucial for controlling immune and inflammatory functions in peripheral tissues. An increase in ACh above a certain threshold can suppress the production of pro-inflammatory cytokines. Acetylcholinesterase (AChE) contributes to regulating ACh levels and, thus, modulates inflammation [92]. In particular, ACh produced by the vagus nerve and/or by peripheral leukocytes [93] can potently modulate several classical immune reactions by activating the α7-nicotinic ACh receptor on the leukocyte membrane, which in turn blocks the nuclear factor kappa B (NF-κB)-mediated production of pro-inflammatory cytokines, such as IL1β and tumor necrosis factor alpha [92]. Because mesenchymal stromal/stem cells carry both nicotinic and muscarinic ACh receptors [94], niche-derived cholinergic signals may play a role in hematopoiesis by regulating proliferation and apoptosis of HSPCs undergoing erythroid and myeloid differentiation [95].

The *ACHE* gene includes multiple putative binding sites for hematopoietic transcription factors. Alternative splicing gives rise to “synaptic” (AChE-S) multimers, which control ACh levels in the brain and muscles, “erythrocyte” (AChE-E) dimers and stress-induced “read-through” (AChE-R) monomers [96]. AChE-R is involved in cell proliferation, whereas AChE-S can be induced during apoptosis [97]. Under stress responses, blood AChE-R undergoes C-terminal cleavage rendering a C-terminal peptide (ARP) of 55 kDa, which promotes the myeloproliferation and thrombopoiesis characteristics of cellular stress [98]. Because ARP functions as a hemopoietic growth factor promoting proliferation of CD34^+^ HSPCs, circulating AChE-R and/or ARP might be involved in directing CD34^+^ HSPCs towards prolonged granulocytosis [96]. Furthermore, *ACHE* has been reported to play a role in hematopoiesis by regulating proliferation, differentiation and apoptosis of erythroid and myeloid progenitors. This might explain, at least in part, the association of perturbations in *ACHE* gene expression with myeloid leukemia [99], particularly after exposure to anticholinesterase insecticides, such as OPs.

*ACHE* is located on chromosome 7q22 within a critical region subject to non-random chromosomal abnormalities. The remarkable abundance of SINEs (short interspersed elements), in particular Alu repeats, in the *ACHE* locus implies exceptional susceptibility to retrotransposition events, which are assisted by the existence of chromosomal breakages. Alu repeats also facilitate unequal crossing-over, altogether contributing to the instability of this region. Chromosomal rearrangements could result in the loss of upstream transcription factor binding sites and, thus, may affect *ACHE* gene expression under stress or exposure to anti-AChE agents. This explains the reported chromosomal aberrations involving 7q22 in leukemic patients [100]. The proximal promoter of the *ACHE* gene contains consensus motifs for the leukemia-associated factor AML1/Runx1 and c-fos, a transcription factor known to regulate *ACHE* gene expression under stress [101]. Hence, the loss of DNA on chromosome 7 may play a significant role in AML [95,96,97,98,99,100,101,102]. Furthermore, a study of 1880 children with ALL reported that 4% of them had DNA losses involving chromosome 7 [103].

A pivotal role of AChE has been suggested in apoptosis. While the 55-kDa AChE protein is selectively induced during apoptosis, its suppression inhibits apoptosome formation and rescues cells from apoptosis [104]. The 55-kDa AChE protein is negatively regulated by the activation of the phosphatidylinositol-3 kinase (PI3K)/protein kinase B (Akt) pathway [104,105]. This signaling cascade is crucial to cell cycle progression, transcription, translation, differentiation, apoptosis, motility and metabolism [106]. The decrease in AChE activity and the consequent increased level of ACh could cause cholinergic overstimulation and enhance cell proliferation in lung cancer [97]; however, whether a similar effect can occur in leukemogenesis is unknown. On the other hand, AChE can hydrolyze lipid peroxides, raising the possibility that a reduction in enzyme activity increases oxidative stress and cellular damage [97].

## 7. Conclusions

Overall, there is sustained epidemiological evidence to suggest a risk of pediatric leukemia upon exposure (*in utero* and/or after birth) to some classes of pesticides, but scientific/mechanistic studies to definitively support this association are lacking. Pesticides may induce topoisomerase II inhibition or generation of oxidative stress, consistently leading to misrepaired DNA cleavage and further chromosomal aberrations in HSPCs. This early molecular event might be sufficient for triggering infant leukemia, but not childhood leukemia, which requires further postnatal events for overt disease. The combination of epidemiological and case-based genomic studies together with cell biology analyses would be useful to elucidate the etiology of pediatric leukemia. In particular, this approach would help to better understand the biological and genetic evidence that is pertinent to the mechanisms by which pesticides might impact on the risk of pediatric leukemia.
